# Temporal and Reciprocal Relations Between Worry and Rumination Among Subgroups of Metacognitive Beliefs

**DOI:** 10.3389/fpsyg.2020.551503

**Published:** 2020-09-11

**Authors:** Frederick Anyan, Roxanna Morote, Odin Hjemdal

**Affiliations:** Department of Psychology, Norwegian University of Science and Technology, Trondheim, Norway

**Keywords:** metacognitive beliefs, worry, rumination, metacognitive theory, autoregressive cross-lagged modeling

## Abstract

Metacognitive theory provides strong foundation for hypothesizing relations between worry and rumination among subgroups of metacognitive beliefs. However, empirical exploration of prospective and reciprocal relations between worry and rumination are lacking. This study investigated the stability and relations between worry and rumination to better understand how they influence each other over time, and how different levels of metacognitive beliefs affect relations between (i) initial and future worry, and initial and future rumination, and (ii) the cross-lag relations between worry and rumination. Overall, 482 (Females = 63%) participants (Mean age = 26 years) participated in a two-wave data collection and completed the Metacognition Questionnaire (MCQ-30), the Ruminative Response Scale and the Penn State Worry Questionnaire (PSWQ). A multigroup two-wave autoregressive cross-lagged model was estimated. Multigroup autoregression analyses revealed that independent of participants being in the high or low metacognition group, initial levels of worry predicted future levels of worry, as was the case for rumination. Multigroup cross-lagged analyses revealed that initial levels of worry did not predict future levels of rumination in both high and low levels of metacognitions. However, initial rumination predicted future levels of worry in the high metacognitions group, which was not the case for the low metacognitions group. Thus, high levels of metacognitions do not only strengthen the relation between both present and future worry, present and future rumination, but also present rumination with future worry. This finding may imply that those with rumination related conditions at present are more likely in the future to show both rumination and worry related conditions. Conversely, those with worry related conditions show future worry related conditions. These findings may have implications for a clinical sample regarding the high complexity of rumination conditions that may proceed with multifinality causal pathways especially for individuals with high levels of metacognitions. This complexity may be a possible explanation for the limited success in other traditional treatment of rumination related conditions and the relatively high relapse rates for such conditions in clinical samples.

## Introduction

Metacognitive beliefs provide the supporting framework for monitoring, evaluating and interpreting repetitive negative thinking such as worry, and rumination. According to the Self-regulatory Executive Function (S-REF) model (Wells and Matthews, 1994, 1996; Wells, 2009), metacognitive beliefs are the knowledge base, and information processing system in which if inflexible, repetitive, and maladaptive thinking persist, it becomes central for developing and maintaining emotional and mental health problems. For example, anxious, uncontrollable, and excessive worry operationalized by the Penn State Worry Questionnaire (Meyer et al., 1990) and depressive rumination operationalized by the Ruminative Response Scale (Nolen-Hoeksema and Morrow, 1991) are viewed by the S-REF as part of the cognitive attentional syndromes (CAS), which is involved in producing counterproductive effects, thereby developing and maintaining emotional and mental health problems (Papageorgiou and Wells, 2003). The metacognitive model postulate that metacognitive beliefs determine whether an individual’s worry or rumination is maintained and exacerbated (Papageorgiou and Wells, 2003). The purpose of the present study was to examine the prospective and reciprocal relations between worry and rumination among subgroups of low and high levels of metacognitive beliefs, measured by the metacognition questionnaire (MCQ – 30; Wells and Cartwright-Hatton, 2004).

Worry is a cognitive activity and a thinking style defined as repetitive negative thinking about future events (Borkovec, 1994). When one worries, it is mainly due to uncertainty about anticipated threats, which could result in underestimation of personal agency, abilities and controllability of future events (Papageorgiou and Wells, 2001b; Nolen-Hoeksema et al., 2008). An individual may also worry as result of the implications that an uncontrollable past event can have for the future (Nolen-Hoeksema et al., 2008). In the Response Styles Theory (Nolen-Hoeksema, 1991), rumination was thought of as a maladaptive cognitive activity and a response style. It was described as self-focused behaviors and repetitive thinking about negative feelings, its causes and outcomes (Nolen-Hoeksema, 1991). In a later revision, Nolen-Hoeksema et al. (2008) has defined rumination as a process of repetitive negative thinking rather than the specific content of negative thinking. Worry and rumination highly correlate and share several similar features such as abstract thinking style and uncontrollable repetitive negative thinking (Nolen-Hoeksema et al., 2008). Still, many features of worry and rumination are distinguishable. Worry tends to be future-oriented, focusing on anticipated threats whereas rumination tends to be past- or present-oriented, focusing on self-worth, meaning, and themes of loss and failure (Papageorgiou and Wells, 2001b; Nolen-Hoeksema et al., 2008).

Metacognitive processes contribute to maintain and strengthen worry or rumination for developing emotional and subsequent mental health problems more than the content of worry or rumination (Wells, 2009). Metacognitive processes are operationalized by the MCQ-30; Wells and Cartwright-Hatton, 2004), which was adapted from the original MCQ-65 (Cartwright-Hatton and Wells, 1997). The MCQ-30 retained the five factors contained in the original scale, comprising, (i) positive beliefs about worry (e.g., *“Worrying helps me cope”*), (ii) negative beliefs about uncontrollability and danger (e.g., *“When I start worrying I cannot stop”*), (iii) lack of cognitive confidence (e.g., *“I have little confidence in my memory for places”*), (iv) need to control thoughts (e.g., *“Not being able to control my thoughts is a sign of weakness”*), and (v) cognitive self-consciousness (e.g., *“I pay close attention to the way my mind works”*) (for the full scale please consult Wells, 2009). For example, individuals may hold positive metacognitive beliefs about worry that suggest potential benefits of worrying, thereby increasing the use of worry as coping strategy for uncertainty about future events. However, prolonged use of worry as coping strategy may be accompanied by or gradually degenerate into other negative metacognitive beliefs about worry related to, for example, the uncontrollability of thought processing. Thus, the combination of positive and negative beliefs about using worry develop and maintain emotional and mental health problems (Wells, 2009). Similarly, positive beliefs about rumination (e.g., “Ruminating about the past helps me to prevent future mistakes and failures”) can motivate individuals to sustain rumination (Papageorgiou and Wells, 2001b). Prolong processing of negative beliefs about rumination (e.g., “Ruminating about my problems is uncontrollable”) can lead to increased use of maladaptive coping strategies and justification for withdrawal and inactivity due to hopelessness and uncontrollability (Papageorgiou and Wells, 2001b; Nolen-Hoeksema et al., 2008). Wells (2009) provides a detailed description and application of all five metacognitive components.

Metacognitive therapy (MCT) was developed to eliminate the CAS by addressing metacognitive processing in negative thinking that contributes to emotional and mental health problems (Wells, 2009), and has since received support from several recent clinical studies (e.g., Wells et al., 2009; Callesen et al., 2014,2020; Jordan et al., 2014; Dammen et al., 2015; Papageorgiou and Wells, 2015; Hagen et al., 2017; Nordahl et al., 2018; Hjemdal et al., 2019; Solem et al., 2019) that underline the importance of metacognitive beliefs. Two meta-analytic reviews have examined the efficacy of metacognitive therapy for anxiety and depression (Normann et al., 2014), and more recently for various disorders including, depression, generalized anxiety, post-traumatic stress, a transdiagnostic sample and other psychological complaints (Normann and Morina, 2018). Hamonniere and Varescon (2018) through a systematic review have also highlighted the central role of metacognitive beliefs in addictive behaviors (e.g., alcohol use, nicotine use, gambling, online gaming, and problematic internet use).

The results have unanimously articulated MCT as an effective treatment for various psychological disorders, showing the strongest evidence for anxiety and depression. Furthermore, results show that MCT may be superior to other psychotherapies, including cognitive behavioral interventions. Recently, it has been found that MCT was superior to CBT at post-treatment and follow-up on depression symptoms measured using BDI-II. More specifically, 74% of patients in MCT compared with 52% in CBT met formal criteria for recovery on the BDI-II at post-treatment [odds-ratio = 2.42 (1.20–4.92), *p* = 0.014]. At follow-up the proportions were 74% compared to 56% recovery [odds-ratio = 2.19 (1.05–4.54), *p* = 0.036]. The MCT therapy increases awareness of metacognitive processes, reduces rumination, worry, and threat monitoring as well as addressing maladaptive coping strategies. MCT also facilitates control and attentional flexibility as well as modifies positive and negative metacognitive beliefs. For example, a study by Hjemdal et al. (2019) compared a waiting condition and MCT for unipolar depression (*N* = 39) and found that patients who recovered showed lager reductions in negative and positive beliefs about rumination, negative metacognitions, and worrying than patients who did not recover. Nordahl et al. (2018) compared MCT and CBT for long-term GAD patients, randomizing them into CBT (*n* = 28), MCT (*n* = 32), and waiting control (*n* = 21). Although both CBT and MCT were effective, MCT was more effective and led to significantly higher recovery rate (65 vs. 38%), maintained at 2-year follow-up. These studies may be taken as examples of the importance of addressing metacognitive beliefs in treatments to reduce emotional and mental health problems.

Other studies also highlight the importance of metacognitive beliefs, worry and rumination in contributing to emotional and mental health problems. Ryum et al. (2017) investigated whether metacognitive beliefs predicted the development of anxiety over and above worry (*N* = 190) and found that metacognitive beliefs predicted the development of anxiety over 7-month period, even when controlling for worry, but their interaction effect was not significant. Although, the results suggest no moderation between worry and metacognitive beliefs, they also open further possibilities to extending and testing differential effect of metacognitive beliefs on worry and rumination. If different subgroups of metacognitive beliefs show differences in the way metacognitions affect worry and/or rumination, this can help us to understand how different levels of metacognitive beliefs are involved in processing worry and rumination, prospectively and reciprocally. The contribution of worry and rumination in mental health problems have also been examined. Roelofs et al. (2008) found that rumination and worry separately contributed to anxiety and depression in a sample of clinically depressed adults (*N* = 198). However, when both rumination and worry were entered simultaneously, the effect of rumination remained, but not worry. When analyzing two subcomponents of rumination, which were brooding and reflection, only the brooding subcomponent contributed to anxiety and depression whereas the reflection subcomponent contributed to anxiety.

**FIGURE 1:**
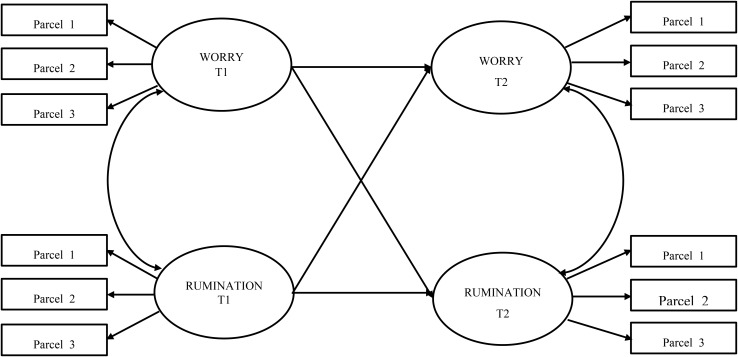
A conceptual path diagram of the two-wave autoregressive cross-lagged model.

Two central contributions from the studies reviewed are relevant for the present study. Firstly, there is documented evidence that metacognitive beliefs may indeed serve as the supporting framework that determines whether an individual’s worry or rumination is maintained and exacerbated, leading to emotional and mental health problems. Secondly, initial research indicates that individuals with high vulnerability in metacognitive beliefs might be associated with higher rumination than worry. Similarly, it can be expected that brooding in combination with high metacognitive beliefs poses a higher risk mechanism than other subcomponents of rumination. Overall, there is strong theoretical foundation for hypothesizing the relations between different levels of metacognitive beliefs, worry and rumination, based on the Metacognitive theory (Wells, 2009). The current study empirically explores these relations to advance potential practical and applied contributions. This study examined whether individual differences in worry and rumination among low and high subgroups of metacognitive beliefs are relatively stable over a 3-month period using autoregressive modeling. Additionally, prospective and reciprocal relations between worry and rumination were examined using cross-lagged modeling. This can help us to understand how different levels of metacognitive beliefs affect relations between (i) initial and future worry, initial and future rumination, and (ii) the prospective, reciprocal relations between worry and rumination. A conceptual path diagram of the two-wave autoregressive cross-lagged model estimated in the present study is displayed in Figure 1.

The present study is conducted among a non-clinical sample, but it may offer some empirical importance, as a first step towards furthering the understanding of how metacognitive beliefs influence temporal and reciprocal relations between worry and rumination. Two main hypotheses were tested in this study. The Metacognitive theory (MCT; Wells and Matthews, 1994, 1996; Wells, 2009), and other controlled randomized (e.g., Nordahl et al., 2018; Hjemdal et al., 2019) and longitudinal (Ryum et al., 2017) studies support worry and rumination as central components of the CAS despite being different in terms of their content, time orientation, processing and metacognitive dimensions (Papageorgiou and Wells, 2001a,b; Nolen-Hoeksema et al., 2008). In MCT, worry and rumination are viewed as repetitive, sustained negative thinking that is associated with more negative thinking and beliefs, producing counterproductive effects that develop and maintain emotional and mental health problems (Papageorgiou and Wells, 2003; Wells, 2009). Thus, it was expected that present worry and rumination would be associated with more worry and rumination over time.

i Autoregressive effects tested the hypothesis that worry at T1 would predict the levels of worry at T2, and rumination at T1 would predict the levels of rumination at T2.

Metacognitive theory provides strong foundation for hypothesizing relations between worry and rumination among clinical samples, but this has not been investigated in subgroups with different levels of metacognitions. According to the Metacognitive theory, some people can successfully respond to the patterns of negative thoughts and beliefs arising from worry and rumination. Other people whose metacognitive processing locks them into prolonged and recurrent patterns of negative thoughts and beliefs develop and maintain emotional and mental health problems (Wells, 2009). Rumination and worry are both viewed as central components of the CAS, but rumination has been found to be a more prominent risk factor than worry (e.g., Roelofs et al., 2008). Thus, it was expected that individuals with higher vulnerability in metacognitive beliefs might be associated with higher rumination than worry.

ii Cross-lagged effects tested the hypothesis that in (combination with) the high metacognition subgroup, rumination at T1 would predict both rumination at T2 and worry at T2. We did not expect worry at T1 to predict rumination at T2.

## Materials and Methods

### Participants and Procedure

Students at the Norwegian University of Science and Technology were invited to take part in the study at two different times, separated by 3 months. Four hundred and eighty-two participants responded to the survey. One hundred and ninety-nine responded in only Time 1 (Mean age = 25.45 years; Females = 127), and 42 students in only Time 2 (Mean age = 25.29 years; Females = 22). Two hundred and forty-one responded in both T1 and T2 (Mean age = 26 years; Females = 155). The project was approved by the Norwegian Ethics committee 2016/339.

### Instruments

#### Worry

The Penn State Worry Questionnaire (PSWQ; Meyer et al., 1990) was used to assess worry. The PSWQ has 16 items that assess the degree to which individuals typically perseverate about upcoming life events, rated on 1–5 Likert type scale (e.g., “My worries overwhelm me”). The PSWQ has been observed to have high internal consistency as well as test–retest reliability (Meyer et al., 1990). The PSWQ has been translated to Norwegian (e.g., Pallesen et al., 2006) and found to have adequate psychometric properties in terms of reliability and validity. In this study, Cronbach’s alpha (α) was (αT1 = 0.942; αT2 = 0.946).

#### Rumination

The Ruminative Response Scale (RRS; Nolen-Hoeksema and Morrow, 1991) is a 22-item self-report questionnaire assessing responses to depressed mood (e.g., Why do I have problems other people don’t have?”). Higher scores indicate higher levels of rumination. Psychometric properties with Cronbach’s alphas have been reported between 0.88 and 0.92 (Luminet, 2004). In this study, Cronbach’s alpha (α) was (αT1 = 0.929; αT2 = 0.936). The RRS has already been used in Norway (Hjemdal et al., 2019).

#### Metacognitive Beliefs

The Metacognitions Questionnaire-30 (MCQ-30; Wells and Cartwright-Hatton, 2004) is a generic questionnaire used to assess dysfunctional metacognitive beliefs according to metacognitive theory. Each item is rated on a 4 Likert-type scale. The MCQ-30 consists of five subscales namely, lack of cognitive confidence, positive beliefs about worry, cognitive self-consciousness, negative beliefs about uncontrollability and danger, and need to control thoughts. Example items include “Worrying helps me to solve problems” and “When I start worrying, I cannot stop.” High scores indicate more dysfunctional metacognitive beliefs. The MCQ has been translated to Norwegian and demonstrated good psychometric properties including good internal consistency, concurrent- and convergent validity (Grøtte et al., 2016). In this study, Cronbach’s alpha (α) was (αT1 = 0.897; αT2 = 0.901).

### Statistical Analyses

Basic correlation analyses and group mean difference tests were performed in SPSS version 25. All other analyses were performed in Mplus version 7.4 (Muthén and Muthén, 1998–2012). We used robust full-information maximum likelihood (MLR) due to missing values and non-normality. In all analyses, autocorrelations in indicator uniqueness were freely estimated, assuming that indicator-specific variances are temporally stable. This also accounts for consistency in indicator variance and captures methodological biases within a measure such as response bias among participants (Wickrama et al., 2016). In accordance with recommendations by Selig and Little (2012), as first step, prior to estimating the structural model for the prospective relations between Worry and Rumination, the measurement model of the two latent variables (i.e., Worry and Rumination) were tested in a longitudinal measurement invariance framework, where each item loaded onto its respective factor and loadings were constrained over time. Three parcels were created for worry by computing the mean of 5, 5, and 6 items. Five items composed of negatively worded items (i.e., items 1, 3, 8, 10, 11), and the rest 11 positively worded items were divided into a 6-item parcel (i.e., items 2, 4, 5, 6, 7, 9) and a 5-item parcel (i.e., items 12, 13, 14, 15, 16). Parceling conceptually related items reduces method effects associated with individual items and random errors while increasing reliability of the structural model, hence, it is considered acceptable when conducting latent variable SEM with multiple indicators (Little et al., 2002; Wang and Wang, 2019). Similarly, three parcels were created for rumination using items that assess brooding (i.e., items 5, 10, 13, 15, 16), reflection (i.e., items 7, 11, 12, 20, 21), and depressive rumination (i.e., items 1, 2, 3, 4, 6, 8, 9, 14, 17, 18, 19, 22), as the indicators of rumination latent variable. Similar approaches have been used by Selig and Little (2012) and Segel-Karpas and Ayalon (2019).

As second step, we tested a multigroup autoregressive cross-lagged panel model of worry and rumination among low and high subgroups of metacognitive beliefs. Participants were split at the mean into low and high metacognitive beliefs subgroups. Each latent variable was allowed to predict subsequent follow-up assessment of itself, measuring the stability of individual differences in the construct from one occasion to the next. Cross-lagged effects were estimated, controlling for the previous level of the construct being predicted. Thus, when worry at Time 2 was predicted by rumination at T1, worry at T1 was controlled to rule out the possibility that the cross-lagged effect is simply due to correlations between worry and rumination at Time 1. Age, gender (1 = Males; 2 = Females) and years of education were added as time invariant control variables. Model fit was evaluated with the following indices: Standardized Root Mean Square Residual (SRMR) (Browne and Cudeck, 1993) and Root Mean Square Error of Approximation (RMSEA) (Hu and Bentler, 1999) values less than 0.08 and values equal to or less than 0.06 (upper 90% CI close to or <0.08), respectively, a Comparative Fit Index (CFI) and a non-Normed Fit index (NNFI; aka TLI) greater than 0.95 (Hu and Bentler, 1999).

## Results

### Preliminary and Attrition Analyses

Table 1 displays the means, standard deviations and correlations among variables. Preliminary comparisons were conducted between participants who completed only one wave (i.e., T1 or T2) and those who completed both waves (i.e., T1 and T2). Significant differences were found in years of education for participants who completed T1 (*M* = 16.08) only and those who completed both waves [*M* = 16.76), *t*(438) = −2.541, *p* < 0.05]. Significant mean differences were found when comparing the high and low metacognitive beliefs subgroups for age *t*(431) = 2.905, *p* < 0.01, worry *t*(429) = −12.461, *p* < 0.001, and rumination *t*(427) = −12.212, *p* < 0.001 but not years of education.

**TABLE 1:** Descriptive statistics and correlations for variables.

		High		Low								
Variable		*M*	*SD*	*M*	*SD*	1	2	3	4	5	6	7
1	Age	24.730	4.364	26.36	7.212	1	–0.060	0.062	–0.086	–0.098	–0.122	–0.158
2	Gender					–0.029	1	0.007	0.325**	0.368**	0.256**	0.252**
3	Years of education	16.260	2.813	16.580	2.830	0.383**	0.039	1	–0.040	–0.053	–0.065	–0.003
4	Worry_T1	3.371	0.868	2.411	0.687	–0.031	0.405**	–0.010	1	0.873**	0.464**	0.414**
5	Worry_T2	3.375	0.774	2.364	0.711	–0.019	0.290**	0.060	0.678**	1	0.499**	0.537**
6	Rumination_T1	2.210	0.541	1.616	0.460	–0.053	0.283**	–0.064	0.473**	0.486**	1	0.766**
7	Rumination_T2	2.194	0.549	1.622	0.480	–0.075	0.270**	–0.078	0.164	0.480**	0.658**	1

**p < 0.01. Correlation coefficients are shown below the diagonal for High metacognition (Mean/SD: Time 1 = 65.35/9.41; Time 2 = 61.81/10.43) and above the diagonal for Low metacognition (Mean/SD: Time 1 = 43.98/5.978; Time 2 = 43.66/8.99).

### Autoregressive and Cross-Lagged Analyses

**FIGURE 2:**
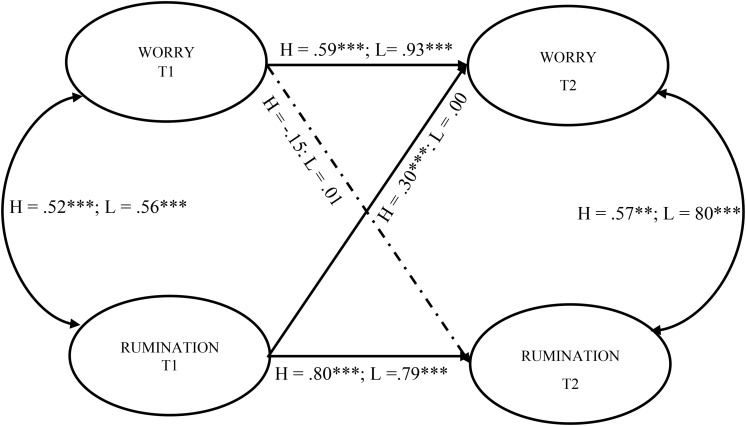
Results from the multigroup two-wave autoregressive cross-lagged model. H, Standardized estimates for High metacognition subgroup; L, Standardized estimate for low metacognition subgroup. Dash-dot line show non-significant relation in both groups. ^∗∗^*p* < 0.01; ^∗∗∗^*p* < 0.001.

The overall model (combined sample) showed good fit to the data (*χ^2^* = 127.909, *df* = 66, *p* < 0.001; SRMR = 0.042; RMSEA = 0.046 [90% CI = 0.034, 0.058]; CFI = 0.981; TLI = 0.970). Autoregressive effects in the overall model were statistically significant (Worry: Standardized *β* = 0.745, *p* < 0.001; Rumination: *β* = 0.849, *p* < 0.001), with significant cross-lagged effect of rumination on worry (*β* = 0.190, *p* < 0.05). Since model fit in the combined sample was good, we proceeded to test the model in the subgroups. The fit of the multigroup model (*χ^2^* = 239.027, *df* = 128, *p* < 0.001; SRMR = 0.070; RMSEA = 0.053 [90% CI = 0.041, 0.066]; CFI = 0.965; TLI = 0.952) was also good. Figure 2 shows the results for the multigroup two-wave autoregressive cross-lagged model of worry and rumination. As expected, autoregressive effects of worry and rumination were statistically significant in both subgroups. Detailed results of the multigroup two-wave autoregressive cross-lagged analyses are displayed in Table 2.

**TABLE 2:** Model Parameter Estimates from the multigroup two-wave, autoregressive cross-lagged analyses.

Subgroups metacognition	High (*n* = 186)				Low (*n* = 247)			
Effects	*B*	*S. E*	*p*	*beta*	*B*	*S. E*	*p*	*Beta*
Worry_1_ → Worry_2_	**0.614**	**0**.**128**	**0**.**000**	**0.586**	**0.961**	**0**.**109**	**0**.**000**	**0.927**
Rumination_1_ → Rumination_2_	**0.880**	**0**.**122**	**0**.**000**	**0.804**	**0.860**	**0**.**132**	**0**.**000**	**0.793**
Worry_1_ → Rumination_2_	–0.133	0.079	0.089	–0.154	0.009	0.104	0.932	0.009
Rumination_1_ → Worry_2_	**0.395**	**0**.**168**	**0**.**000**	**0.299**	–0.004	0.105	0.968	–0.004
Worry_1_ ↔ Rumination_1_	**0.137**	**0**.**027**	**0**.**000**	**0.520**	**0.098**	**0**.**016**	**0**.**000**	**0.563**
Worry_2_ ↔ Rumination_2_	**0.077**	**0**.**023**	**0**.**001**	**0.574**	**0.038**	**0**.**009**	**0**.**000**	**0.797**
**Worry_1_**								
Age →	–0.008	0.010	0.429	–0.057	–0.003	0.004	0.423	–0.052
Gender →	**0.514**	**0**.**098**	**0**.**000**	**0.381**	**0.313**	**0**.**064**	**0**.**000**	**0.323**
Years of education →	–0.001	0.016	0.944	–0.005	–0.008	0.010	0.384	–0.050
**Worry_2_**								
Age →	–0.004	0.007	0.614	–0.024	0.000	0.004	0.908	0.007
Gender →	–0.014	0.116	0.902	–0.010	0.010	0.051	0.841	0.010
Years of education →	–0.018	0.018	0.326	–0.076	–0.008	0.009	0.350	–0.047
**Rumination_1_**								
Age →	–0.001	0.008	0.874	–0.011	–0.006	0.003	0.050	–0.106
Gender →	**0.368**	**0**.**078**	**0**.**000**	**0.345**	**0.237**	**0**.**054**	**0**.**000**	**0.282**
Years of education →	–0.021	0.014	0.118	–0.121	–0.012	0.011	0.264	–0.084
**Rumination_2_**								
Age →	–0.004	0.007	0.567	–0.032	–0.002	0.004	0.580	–0.036
Gender →	0.046	0.099	0.641	0.039	–0.004	0.056	0.941	–0.005
Years of education →	–0.025	0.017	0.129	–0.131	0.014	0.009	0.137	0.089

**Subscripts denote time. Statistically significant paths are shown in boldface. Forty-nine participants were not included for analyses due to missing data.**

For the high subgroup, only the cross-lagged effect of rumination at T1 on worry at T2 was statistically significant, accounting for over half of the variance in worry after 3 months (*R*^2^ = 64.6%). Consequently, the subcomponents of rumination were further explored in this group to examine how the different subcomponents of rumination are prospectively related to worry. Only the model with the brooding subcomponent (*χ^2^* = 177.088, *df* = 126, *p* < 0.001; SRMR = 0.062; RMSEA = 0.047 [90% CI = 0.029, 0.062]; CFI = 0.954; TLI = 0.939) showed statistically significant cross-lagged effect on worry (Standardized β = 0.251, *p* < 0.05). Autoregressive effects were statistically significant (Worry: β = 0.624, *p* < 0.001; Brooding: β = 0.883, *p* < 0.001). Finally, among both subgroups, females were statistically significantly associated with current worry and rumination, but not males. Gender differences accounted for (Female: High subgroup: 15%; Low subgroup: 11.2%) variance in current worry and (Female: High subgroup: 13.2%; Low subgroup: 10.2%) variance in current rumination.

## Discussion

This study investigated prospective and reciprocal relations between worry and rumination among subgroups of low and high metacognitive beliefs over a 3-month period. To our knowledge, this study is the first to provide prospective and reciprocal evidence of the relations between worry and rumination among low and high subgroups of metacognitive beliefs. Firstly, results showed positive longitudinal pairwise correlations between worry and rumination in both subgroups. As the intercorrelations were generally moderate, the results suggest that worry and rumination are associated, but also contain unique aspects as constructs. The results show support for previous findings regarding the phenomenology of worry and rumination. Experiences of worry and rumination have several shared features that show similarities in the two constructs (Papageorgiou and Wells, 2001b; Nolen-Hoeksema et al., 2008). Longitudinal positive pairwise correlations between worry and rumination is consistent with the conceptualizations of worry and rumination as both involving shared features namely, unproductive, abstract over-general thinking style, cognitive inflexibility in switching attention, and uncontrollable repetitive negative thinking (Papageorgiou and Wells, 2001a; Nolen-Hoeksema et al., 2008). Moderate intercorrelations show that worry and rumination are not too highly overlapping and thus, differ in important ways such as in time orientation; worry being associated with anticipated threats of future events, and rumination being associated with negative thinking about self-worth, loss and failures concerning the self in past and current events (Nolen-Hoeksema et al., 2008). Anticipation of future threats in worry may not always develop into mental health problems as described by Sweeny and Dooley (2017), arguing that worry might have motivational and emotional benefits. It is possible that the motivational and emotional benefits of worry explain why some people do not lock attention onto unhelpful processes of worry.

Secondly, in both subgroups, worry and rumination were concurrently significantly related, and their autoregressive effects were significant. This is consistent with the first hypothesis as it was found that independent of participants being in the high or low metacognition subgroup, initial levels of worry predicted future levels of worry, as was the case for rumination. Autoregressive coefficients for worry and rumination imply that individual differences in worry and rumination are relatively stable over the 3 months between occasions of measurement. Hence, researchers and practitioners should pay attention to the possibility that the effect of metacognitive beliefs for processing worry or rumination might persist along the entire continuum of scores on the metacognition scale. Our first hypothesis was based on the Metacognitive theory (Wells and Matthews, 1994, 1996; Wells, 2009), and other empirical studies (e.g., Ryum et al., 2017; Nordahl et al., 2018; Hjemdal et al., 2019), which have also found that metacognitive beliefs have an effect on worry and rumination, and thus altering metacognitive beliefs can help to reduce worry and rumination.

Thirdly, consistent with the second hypothesis, the main finding was that initial levels of worry did not predict future levels of rumination in both high and low levels of metacognitions. However, for the group with high levels of metacognitions, initial rumination predicted future levels of worry, which was not the case for the low metacognitions group. Thus, high levels of metacognitions do not only strengthen the relation between both present and future worry, present and future rumination, but also present rumination with future worry. This finding may imply that those with rumination related conditions at present are more likely in the future to show both rumination and worry related conditions. Conversely, those with worry related conditions show future worry related conditions. This finding is very interesting as it indicates a potentially higher degree of complexity for rumination conditions and points toward multifinality causal pathways, especially for individuals with high levels of metacognitions. This complexity may be a possible explanation for the limited success in other traditional treatment of rumination related conditions and the relatively high relapse rates for such conditions. Rumination is a prominent feature of depression and several studies have established robust evidence for the relation between rumination and greater severity and duration of depression (for review, see Nolen-Hoeksema et al., 2008), rumination, and recurrent depression (Marchetti et al., 2012), and more recently between rumination and increases in the risk of future depressive relapse, suggesting that even after the depressive episode has gone away, continuing rumination can still predict recurrence of depression (Figueroa et al., 2019). Figueroa et al. (2019) investigated cognitive flexibility and rumination in patients whose depression had remitted and tested whether these factors predicted time to depressive recurrence. The authors found that rumination, and its brooding subcomponent, predicted recurrence, which is in accordance with MCT theory (Wells, 2009).

In addition to the main findings, when analyzing rumination subcomponents, initial brooding predicted future worry even when controlling for initial worry among the high subgroup. Previous research findings have also shown that rumination or its brooding subcomponents contributes more than worry as risk factors for increasing the probability of a disorder (Roelofs et al., 2008), that makes rumination a prominent risk factor. The current study found that high vulnerability in metacognitive processing represents a more generalized risk mechanism that may amplify the effects of rumination more than worry in the current sample.

Finally, the varying results in the subgroups also imply that metacognitive beliefs moderate the effect of rumination on worry, but not worry on rumination. This is consistent with the previous finding that no moderation effect existed between worry and metacognitive beliefs (Ryum et al., 2017), although the previous study investigated worry and metacognitive beliefs in predicting anxiety. Overall, asserting that qualitative differences exist from low to high levels of metacognitions in the relations between rumination and worry is supported. Thus, indicating this may be an issue to consider in targeted screening, treatment and follow-up, because of the qualitative differences in the temporal relation between metacognitive processes, worry, and rumination. These results bring into relief the prospective and reciprocal relations between worry and rumination in high levels of metacognitive processing.

Strengths of the present study include using a longitudinal dataset to investigate prospective and reciprocal relations among variables and the partitioning of interindividual variability (i.e., individual differences) to investigate stability across time points by using the autoregressive cross-lagged model. Still, the present study has some limitations. There was floor effect in the scores of worry and rumination for participants in the low metacognition subgroup. Thus, the results should be interpreted with caution as floor effects restrict variance in scores and can potentially mask the effect that metacognition has on the relation between worry and rumination. Differences in how the subcomponents of metacognitive beliefs may influence worry and rumination were not investigated. Future studies are needed to clarify the relation between worry and rumination for individuals scoring differently on the different subcomponents of metacognitive beliefs. Selig and Little (2012) argued that potential limitations when using panel models such as the autoregressive cross-lagged model is the lack of explicit theory of change and weak causal claims. Thus, panel models fail to incorporate intraindividual change, or joint intra- and inter-individual change that unambiguously describe how developmental processes unfolds over time thereby greatly limiting its application by developmental scientists. For example, while it has been found that individual differences in worry and rumination are relatively stable over a 3-month period. The results do not tell us about a more subtle and important change related to within-person effects. Future studies are recommended to use research designs and analyses in latent curve models of stability and change that incorporate the joint intra- and inter-individual change that can purely disaggregate between-person and reciprocal, prospective within-person components of the relations between two or more variables such as the Random Intercepts Crossed-lagged Panel Model (RI-CLPM; Hamaker et al., 2015) or the Latent Curve Model with Structured Residuals (LCM-SR; Curran et al., 2014).

Finally, regarding the time when hypothesized autoregressive or cross-lagged effects may be significant or not significant makes the role of time and the choice of time lag between observations important for developmental applications. In the present study, it was assumed that the cross-lagged effect between worry and rumination occurred at the same time; at 3 months later. This introduces a limitation as intuitively one can think that cross-lagged effects may occur at different times for different constructs and for different people across the developmental lifespan. Therefore, the assumption that the time lag for the relations between worry and rumination will occur simultaneously at 3 months, among low and high subgroups of metacognitive beliefs may be untenable. Nonetheless, results in the present study add to the knowledge about temporal stability and reciprocal relations between worry and rumination across different individuals and levels of metacognitive processing in a normal sample. Future studies should explore whether these temporal relations are similar in clinical samples and for patients that receive psychological therapy.

## Data Availability Statement

The raw data supporting the conclusions of this article will be made available by the authors, without undue reservation, to any qualified researcher. All data request should be sent to odin.hjemdal@ntnu.no.

## Ethics Statement

The studies involving human participants were reviewed and approved by the Norwegian Ethics committee (2016/339). The patients/participants provided their written informed consent to participate in this study.

## Author Contributions

FA contributed to the conceptualization of study, statistical analyses, interpretation of results, and drafted and made substantive edits and revisions to the manuscript. OH guided the conceptualization of the study design, provided feedback on the interpretation of results, and contributed to revising the manuscript either through direct edits or feedback. RM contributed to revising the manuscript either through direct edits or feedback. All authors contributed to the article and approved the submitted version.

## Conflict of Interest

The authors declare that the research was conducted in the absence of any commercial or financial relationships that could be construed as a potential conflict of interest. The reviewer DK declared a past collaboration with one of the authors OH to the handling Editor.
